# Supplementary material for: Anti-beta2-glycoprotein I IgG antibodies are associated with early-onset cryptogenic ischemic stroke
Source: Eur Stroke J. 2025 Jul 5:23969873251351207. Online ahead of print. doi: 10.1177/23969873251351207 (PMC12228635; doi:10.1177/23969873251351207)
Supplement: sj-docx-1-eso-10.1177_23969873251351207 – Supplemental material for Anti-beta2-glycoprotein I IgG antibodies are associated with early-onset cryptogenic ischemic stroke [file sj-docx-1-eso-10.1177_23969873251351207.docx]

**Supplemental Material**

**Antiphospholipid antibodies and cryptogenic ischemic stroke in young adults**

**Authors:**

Nina Jaakonmäki, MD^1^; Tuukka Helin, MD, PhD^2^; Timea Szanto, MD, PhD^3^; Marialuisa Zedde, MD, PhD^4^; Tomi Sarkanen, MD, PhD^5^; Nicolas Martinez-Majander, MD, PhD^6^; Juha Sinisalo, MD, PhD^7^; Ulla Junttola, MD^8^; Petra Redfors, MD, PhD^9^; Bettina von Sarnowski, MD^10^; Ulrike Waje-Andreassen, MD, PhD^11^; Pauli Ylikotila, MD^12^; Nilufer Yesilot, MD^13^; Kristina Ryliskiene, MD, PhD^14^; Lauri Tulkki, MD^6;^ Laura Amaya Pascasio, MD^15^; Radim Licenik, MD, PhD^16^; Phillip Ferdinand, MBChB, MRCP^17^; Eva Gerdts, MD, PhD^18^; Dalius Jatužis, MD, PhD^14^; Alessandro Pezzini, MD, PhD^19^; Janika Kõrv, MD, PhD^20^; Juha Huhtakangas, MD, PhD^8^; Ana Catarina Fonseca, MD, PhD^21^; Lotta Joutsi-Korhonen, MD, PhD^2^; Hugo ten Cate, MD, PhD^22^; Pekka Jäkälä, MD, PhD^1^; Jukka Putaala, MD, PhD^6^; and the SECRETO Study Group.

**Affiliations:**

^1^Neurocenter Neurology, Kuopio University Hospital, Finland and University of Eastern Finland; ^2^Department of Clinical Chemistry, HUS Diagnostic Center, Helsinki University Hospital and University of Helsinki, Helsinki; ^3^Coagulation Disorders Unit, Department of Hematology, Comprehensive Cancer Centre, Helsinki University Hospital, Helsinki, Finland; ^4^Neurology Unit, Stroke Unit, Azienda Unità Sanitaria Locale – IRCCS di Reggio Emilia, Italy; ^5^Department of Neurology, Tampere University Hospital, Wellbeing Services County of Pirkanmaa, and Faculty of Medicine and Health Technology, Tampere University, Finland; ^6^Department of Neurology, Helsinki University Hospital, and University of Helsinki, Finland; ^7^Cardiology, Helsinki University Hospital and University of Helsinki; ^8^Clinical Neuroscience Research Unit and Department of Neurology, Oulu University Hospital, Finland; ^9^Department of Neurology, Sahlgrenska University of Hospital and Department of Clinical Neuroscience, Institute of Neuroscience and Physiology, Sahlgrenska Academy at University of Gothenburg, Sweden; ^10^Department of Neurology, University Medicine Greifswald, Greifswald, Germany; ^11^Department of Neurology, Haukeland University Hospital, Bergen, Norway; ^12^Department of Neurology, Turku University Hospital and University of Turku, Finland; ^13^Department of Neurology, Istanbul University, Istanbul Faculty of Medicine, Turkey; ^14^Vilnius University, Faculty of Medicine, Institute of Clinical Medicine, Department of Neurology and Neurosurgery, Lithuania; ^15^Department of Neurology, Torrecárdenas University Hospital, University of Almería, Spain; ^16^Acute Stroke Centre, North West Anglia NHS Foundation Trust, UK**;** ^17^Neurosciences, University Hospitals of North Midlands NHS Trust, Stroke-on-Trent, UK; ^18^Department of Heart Disease, Haukeland University Hospital, Bergen, Norway;; ^19^Department of Medicine and Surgery, University of Parma, Stroke Care Program, Department of Emergency, Parma University Hospital, Parma, Italy; ^20^Department of Neurology and Neurosurgery, University of Tartu, Estonia; ^21^Department of Neurology, Hospital de Santa Maria, Institute of Pharmacology and Neurosciences, Faculdade de Medicina, Universidade de Lisboa, Portugal; ^22^Department of Internal Medicine, Maastricht University Medical Center and CARIM School for Cardiovascular Diseases, Maastricht, the Netherlands.

**Table of contents:**

| Name | Title |
| --- | --- |
| Table S1 | Definitions of Traditional and Non-Traditional Risk Factors of Cryptogenic Ischemic Stroke Patients and Stroke-Free Controls |
| Table S2 | Clinical characteristics of young ischemic stroke patients stratified by presence or absence of antiphospholipid antibodies. |
| Table S3 | Frequencies of Borderline and Positive Lupus Anticoagulant Results from Cryptogenic Ischemic Stroke Patients at Baseline and 12 weeks and Healthy Controls at One Time-Point |
| Table S4 | Odds Ratios and 95% Confidence Intervals from Conditional Logistic Regression on the Association of Lupus Anticoagulant with Cryptogenic Ischemic Stroke in Young Adults Despite Anticoagulation. Antiphospholipid Antibodies Present at Either Time-Point in Patients Compared to One Time-Point in Controls. |
| Table S5 | Adjusted Cox Regression Model Results on the Association of Antiphospholipid Antibodies with Cryptogenic Ischemic Stroke in Young Adults, Stratified by Sex. Antiphospholipid Antibodies Present at Both Time-Points. Patients Were Compared to Antiphospholipid Antibodies Present at One Time-Point in Controls. Positive and borderline results were considered |
| Table S6 | Exploratory Subgroup Analyses of Factors that Might Interact with the Association between Lupus Anticoagulant and Early-Onset Cryptogenic Ischemic Stroke at Either Baseline or 12 weeks for Patients and at Baseline for Healthy Controls |
| Table S7 | Exploratory Subgroup Analyses of Factors that Might Interact with the Association between Anti-β2-glycoprotein I Antibodies and Early-Onset Cryptogenic Ischemic Stroke at Either Baseline or 12 weeks for Patients and at Baseline for Healthy Controls |
| Table S8 | Exploratory Subgroup Analyses of Factors that Might Interact with the Association between Anticardiolipin Antibodies and Early-Onset Cryptogenic Ischemic Stroke at Either Baseline or 12 weeks for Patients and at Baseline for Healthy Controls |
| Table S9 | Univariable Analyses of the Association between Antiphospholipid Antibodies and Cryptogenic Ischemic Stroke among Patients Diagnosed with High-Risk Patent Foramen Ovale (PFO) |
| Table S10 | List of SECRETO study centers enrolling participants and investigators involved |
| Figure SI | Flow Chart of Inclusion of Early-Onset Cryptogenic Stroke Study Patients and Healthy Controls Into Present Study |
| References |  |

**Table S1.** Definitions of Traditional and Non-Traditional Risk Factors.

| **Risk Factor** | **Definition** |
| --- | --- |
| Diabetes mellitus | Prior diabetes diagnosis and/or prior antidiabetic medication at the time of stroke |
| History of cardiovascular disease | Any of coronary heart disease, congestive heart failure, or peripheral arterial disease |
| Family history of stroke | Any stroke, 1st degree relative or grandparent |
| History of venous thrombosis | Prior diagnosis of venous thrombosis |
| History of hypertension | Prior hypertension diagnosis (unless strictly pregnancy-related), being on antihypertensive medication at the time of stroke (in control subjects, at the time of their study visit), or having a mean office blood pressure of ≥140/90 mm Hg on two measurements at a baseline study visit (in control subjects, at their study visit). |
| Hypercholesterolemia | Prior diagnosis and/or prior medication at the time of stroke, or at time of baseline visit for controls. |
| Migraine with aura | Assessed with a validated migraine screening protocol.^1^ |
| Cigarette smoking | Smoking at least one cigarette per day on average during the year prior to the index stroke |
| Physical inactivity | Short version of the International Physical Activity Questionnaire,^2^ and it was defined as total metabolic equivalents per week below 1500 |
| Heavy alcohol consumption | > 7 (women) and >14 (men) units per week, or at least an average of two times per month ≥5 units (women) and ≥7 (men) units per instance (binge drinking).^3^ |
| Dietary habits | A modified version of the Mediterranean diet score,^4^ which excluded alcohol intake, as this was assessed separately. |
| Increased waist-to-hip ratio (WHR) | Used to describe abdominal obesity and was defined according to WHO guidelines.^5,6^ |
| Psychosocial stress | A combined measure of general stress at home and at work (permanent or several periods of stress versus no or some periods of stress in the past year) |
| Depression | Feeling sad, blue, or depressed for two or more consecutive weeks in the prior 12 months. |
| Patent foramen ovale (PFO) | Assessed by echocardiography and transcranial Doppler ultrasound (TCD) bubble screen. A PFO with clinical relevance was defined as PFO with high-risk features including an atrial septal aneurysm or a large-sized shunt (≥25 microbubbles crossing the atrial septum in transesophagial echocardiography or detected in TCD bubble screen).^7^ |

|  | Any aPL | | *P* value |
| --- | --- | --- | --- |
|  | Positive  (n=95) | Negative  (n=344) |  |
| Age,y | 39.7 (30.7-46.2) | 40.6 (34.1-45.5) | 0.365 |
| Hypertension | 30 (31.6) | 116 (33.7) | 0.397 |
| Diabetes mellitus | 2 (2.1) | 11 (3.2) | 0.440 |
| Abdominal obesity | 51 (53.7) | 205 (59.6) | 0.180 |
| Cardiovascular disease | 3 (3.2) | 5 (1.5) | 0.239 |
| Excess alcohol use | 12 (12.6) | 50 (14.5) | 0.388 |
| Current smoking | 25 (26.3) | 119 (34.9) | 0.072 |
| Physical inactivity | 22 (23.4) | 93 (27.4) | 0.260 |
| Depression | 36 (37.9) | 102 (29.7) | 0.083 |
| Psychosocial stress | 52 (54.7) | 168 (49.0) | 0.190 |
| Unhealthy diet | 49 (51.6) | 175 (51.0) | 0.508 |
| Use of estrogen* | 12 (12.6) | 39 (11.3) | 0.423 |
| Migraine with aura | 34 (35.8) | 151 (43.9) | 0.096 |
| Family history of stroke | 5 (5.6) | 27 (8.3) | 0.276 |
| Signs of infection when samples taken | 11 (11.8) | 20 (5.9) | 0.051 |
| Laboratory results |  |  |  |
| Hemoglobin | 145.5 (134.3-154.3) | 143.0 (133.3-152.0) | 0.284 |
| Platelet count | 242.50 (199.5-294.5) | 248.5 (201.5-285.0) | 0.309 |
| C-reactive protein | 1.5 (1.5-3.0) | 1.5 (1.5-3.0) | 0.605 |
| Leukocytes | 7.3 (5.7-8.4) | 7.9 (6.2-9.8) | 0.008 |
| Creatinine | 77.0 (60.3-90.0) | 70.0 (56.3-80.0) | 0.003 |
| Alanine aminotransferase | 20.5 (15.0-35.8) | 22.0 (14.0-31.8) | 0.441 |
| Gamma-glutamyl transferase | 20.0 (14.0-32.0) | 19.0 (12.0-32.0) | 0.472 |
| Low-density lipoprotein | 3.1 (2.5-3.5) | 3.0 (2.3-3.6) | 0.287 |
| International normalised ratio | 1.00 (0.9-1.1) | 1.00 (1.0-1.1) | 0.372 |

**Table S2.** Clinical Characteristics of Young Ischemic Stroke Patients Stratified by Presence or Absence of Antiphospholipid Antibodies (aPL)at Baseline or 12 Weeks.

Data are n (%) or median (interquartile range)

Any aPL refers to any of the following antiphospholipid antibodies; anti-beta2

-glycoprotein IgG, anticardiolipin antibodies, lupus anticoagulant.

Positive = at least one of the aPLs positive at baseline or 12 weeks

Negative = all of the aPLs negative at baseline and 12 weeks

*Only female patients included

**Table S3.** Frequencies of Borderline and Positive Lupus Anticoagulant Results from Cryptogenic Ischemic Stroke Patients at Baseline and 12 Weeks and Healthy Controls at One Time-Point.

| **LA result** | **Controls** | **Patients**  **Baseline** | **Patients**  **12 weeks** |
| --- | --- | --- | --- |
| LA negative | 446 (88.7) | 417 (83.1) | 421 (83.7) |
| RVTT-ratio low positive  1.15/1.25-1.39 | 12 (2.4) | 12 (2.4) | 2 (0.4) |
| RVTT-ratio medium positive  1.40-2.0 | 4 (0.8) | 6 (1.2) | 5 (1.0) |
| RVTT-ratio high positive  >2.0 | 0 | 0 | 1 (0.2) |
| APTT-ratio low positive  1.10-1.40 | 30 (6.0) | 16 (3.2) | 5 (1.0) |
| RVTT and APTT both positive | 0 | 7 (1.4) | 3 (0.6) |
| RVTT borderline | 2 (0.4) | 0 | 0 |
| APTT borderline | 1 (0.2) | 0 | 0 |

Data are n (%)

**Table S4.** ORs and 95% CI from Conditional Logistic Regression on the Association of Lupus Anticoagulant with Cryptogenic Ischemic Stroke in Young Adults Despite Anticoagulation. Antiphospholipid Antibodies Present at Either Time-Point in Patients Compared to One Time-Point in Controls.

| **Lupus Anticoagulant (including anticoagulated study subjects)** | **Model adjusted for age and level of education** | **Model adjusted for age, level of education, and vascular risk factors*** | **Model adjusted for age, level of education, vascular risk factors*, and migraine with aura** |
| --- | --- | --- | --- |
| All | 0.37 (0.24-0.59) | 0.39 (0.24-0.64) | 0.42 (0.25-0.69) |
| Men | 0.28 (0.15-0.52) | 0.30 (0.15-0.59) | 0.33 (0.16-0.67) |
| Women | 0.56 (0.28-1.12) | 0.58 (0.26-1.29) | 0.57 (0.27-1.21) |

Data are odds ratio (95% confidence interval).

*Hypertension, diabetes mellitus, hypercholesterolemia, current tobacco smoking, physical inactivity, excessive alcohol use, stress, depression, unhealthy diet, abdominal obesity, and in women, estrogen use. In women, diabetes mellitus and hypercholesterolemia were excluded due to their low frequency.

**Table S5. Adjusted Cox Regression Model Results on the Association of Antiphospholipid Antibodies With Cryptogenic Ischemic Stroke in Young Adults, Stratified By Sex. Antiphospholipid Antibodies Present at Both Time-Points. Patients Were Compared to Antiphospholipid Antibodies Present at One Time-Point in Controls. Positive and Borderline Results were Considered.**

|  | **Model adjusted for age and level of education** | **Model adjusted for age, level of education, and vascular risk factors*** | **Model adjusted for age, level of education, vascular risk factors*, and migraine with aura** |
| --- | --- | --- | --- |
| **All** | | | |
| LA | 0.07 (0.02-0.25) | 0.07 (0.02-0.24) | 0.07 (0.02-0.25) |
| aβ2GPI | 1.13 (0.47-2.70) | 1.53 (0.61-3.88) | 2.65 (0.98-7.15) |
| aCL | 0.82 (0.38-1.74) | 0.57 (0.25-1.29) | 0.68 (0.29-1.62) |
| aβ2GPI and/or aCL | 0.94 (0.51-1.76) | 0.84 (0.43-1.65) | 1.15 (0.56-2.36) |
| Any APL | 0.37 (0.21-0.66) | 0.31 (0.17-0.55) | 0.36 (0.20-0.67) |
| **Men** |  |  |  |
| LA | 0.11 (0.03-0.46) | 0.05 (0.01-0.25) | 0.04 (0.01-0.24) |
| aβ2GPI | 0.71 (0.21-2.37) | 1.09 (0.29-4.14) | 2.80 (0.64-12.31) |
| aCL | 0.55 (0.22-1.34) | 0.35 (0.12-1.00) | 0.42 (0.14-1.28) |
| aβ2GPI and/or aCL | 0.67 (0.30-1.45) | 0.57 (0.23-1.41) | 0.74 (0.28-1.94) |
| Any APL | 0.36 (0.19-0.69) | 0.24 (0.11-0.53) | 0.25 (0.11-0.60) |
| **Women** |  |  |  |
| LA | 0.05 (0.01-0.37) | 0.06 (0.01-0.49) | 0.08 (0.01-0.67) |
| aβ2GPI | 1.91 (0.52-7.06) | 2.41 (0.59-9.88) | 3.83 (0.83-17.67) |
| aCL | NA† | NA† | NA† |
| aβ2GPI and/or aCL | 1.89 (0.61-5.86) | 2.07 (0.60-7.19) | 3.87 (0.99-15.14) |
| Any APL | 0.37 (0.16-0.89) | 0.42 (0.16-1.08) | 0.64 (0.23-1.75) |

Data are odds ratio (95% confidence interval). aPL, Antiphospholipid antibody; LA, lupus anticoagulant; aCL, anticardiolipin antibodies; aβ2GPI, anti-β2 glycoprotein I -glycoprotein I antibodies.

*Hypertension, diabetes mellitus, current tobacco smoking, physical inactivity, excessive alcohol use, stress, depression, unhealthy diet, abdominal obesity, and in women, estrogen-containing contraception use. In women, diabetes mellitus was excluded, due to its low frequency.

†Not applicable due to small frequency

**Table S6. Exploratory Subgroup Analyses of Factors That Might Interact With the Association Between Lupus Anticoagulant and Early-Onset Cryptogenic Ischemic Stroke at Either Baseline or 12 Weeks for Patients and at Baseline for Healthy Controls.**

| **Interaction factor** | **LA** | | **Unadjusted OR (95% CI)** | ***P* for interaction*** |
| --- | --- | --- | --- | --- |
|  | negative | positive |  |  |
| Hypertension |  |  |  | 0.115 |
| No | 266/310 | 30/41 | 0.88 (0.54-1.45) |  |
| Yes | 132/133 | 16/10 | 1.71 (0.76-3.88) |  |
| Current smoking |  |  |  | 0.754 |
| No | 265/371 | 32/45 | 1.03 (0.64-1.65) |  |
| Yes | 130/71 | 14/6 | 1.37 (0.51-3.67) |  |
| Excessive alcohol use |  |  |  | 0.817 |
| No | 341/412 | 41/49 | 1.04 (0.67-1.60) |  |
| Yes | 57/32 | 5/2 | 1.68 (0.32-8.84) |  |
| Abdominal obesity |  |  |  | 0.524 |
| No | 170/241 | 16/26 | 0.93 (0.49-1.76) |  |
| Yes | 228/201 | 30/24 | 1.14 (0.65-2.01) |  |
| Physical inactivity |  |  |  | 0.252 |
| No | 287/349 | 33/35 | 1.18 (0.72-1.94) |  |
| Yes | 106/89 | 12/16 | 0.68 (0.31-1.49) |  |
| Migraine with aura |  |  |  | 0.912 |
| No | 221/372 | 34/45 | 1.31 (0.82-2.10) |  |
| Yes | 177/72 | 12/6 | 0.88 (0.32-2.41) |  |
| Use of estrogen† |  |  |  | 0.072 |
| No | 143/167 | 8/19 | 0.55 (0.24-1.26) |  |
| Yes | 45/39 | 4/1 | 4.33 (0.49-38.70) |  |

Data are number of patients with cryptogenic ischemic stroke/number of controls; LA, lupus anticoagulant.OR, odds ratio; CI, confidence interval

*P-value from logistic regression model, adjusted for age, hypertension, diabetes mellitus, current tobacco smoking, abdominal obesity, physical inactivity, excessive alcohol use, unhealthy diet, stress, depression, and migraine with aura.

†Only female study subjects were considered.

**Table S7. Exploratory Subgroup Analyses of Factors that Might Interact with the Association Between anti-β2-glycoprotein I Antibodies and Early-Onset Cryptogenic Ischemic Stroke at Either Baseline or 12 Weeks for Patients and at Baseline for Healthy Controls.**

| **Interaction factor** | **aβ2GPI** | | **Unadjusted OR (95% CI)** | ***P* for interaction*** |
| --- | --- | --- | --- | --- |
|  | negative | positive |  |  |
| Hypertension |  |  |  | 0.329 |
| No | 250/340 | 36/9 | 5.44 (2.57-11.50) |  |
| Yes | 128/141 | 15/1 | 16.52 (2.15-126.86) |  |
| Current smoking |  |  |  | 0.902 |
| No | 249/404 | 39/10 | 6.33 (3.10-12.90) |  |
| Yes | 126/76 | 12/0 | 7.84 (1.01-61.14) |  |
| Excessive alcohol use |  |  |  | 0.932 |
| No | 321/448 | 46/10 | 6.42 (3.19-12.91) |  |
| Yes | 57/34 | 5/0 | 3.68 (0.43-31.90 |  |
| Abdominal obesity |  |  |  | 0.217 |
| No | 152/261 | 29/4 | 12.45 (4.29-36.09) |  |
| Yes | 226/218 | 22/6 | 3.54 (1.41-8.89) |  |
| Physical inactivity |  |  |  | 0.021 |
| No | 270/376 | 41/7 | 8.16 (3.60-18.46) |  |
| Yes | 102/100 | 10/3 | 3.27 (0.87-12.23) |  |
| Migraine with aura |  |  |  | 0.775 |
| No | 217/402 | 29/9 | 5.97 (2.78-12.84) |  |
| Yes | 161/80 | 22/1 | 10.93 (1.45-82.56) |  |
| Use of estrogen† |  |  |  | 0.043 |
| No | 131/182 | 17/4 | 5.91 (1.94-17.95) |  |
| Yes | 42/41 | 8/0 | 8.79 (1.06-72.49) |  |

Data are number of patients with cryptogenic ischemic stroke/number of controls; aβ2GPI, anti-β2-glycoprotein I antibodies. OR, odds ratio; CI, confidence interval

*P-value from logistic regression model, adjusted for age, hypertension, diabetes mellitus, current tobacco smoking, physical inactivity, excessive alcohol use, unhealthy diet, stress, depression, and migraine with aura.

†Only female study subjects were considered.

**Table S8 Exploratory Subgroup Analyses of Factors That Might Interact with the Association Between Anticardiolipin Antibodies and Early-Onset Cryptogenic Ischemic Stroke at Either Baseline or 12 Weeks for Patients and at Baseline for Healthy Controls.**

| **Interaction factor** | **aCL** | | **Unadjusted OR (95% CI)** | ***P* for interaction*** |
| --- | --- | --- | --- | --- |
|  | negative | Borderline or positive |  |  |
| Hypertension |  |  |  | 0.941 |
| No | 253/337 | 35/11 | 4.24 (2.11-8.51) |  |
| Yes | 122/137 | 21/5 | 4.72 (1.73-12.89) |  |
| Current smoking |  |  |  | 0.226 |
| No | 249/400 | 40/13 | 4.94 (2.59-9.43) |  |
| Yes | 123/73 | 16/3 | 3.17 (0.89-11.23) |  |
| Excessive alcohol use |  |  |  | 0.937 |
| No | 320/441 | 49/16 | 4.22 (2.36-7.56) |  |
| Yes | 55/34 | 7/0 | 4.95 (0.60-41.71) |  |
| Abdominal obesity |  |  |  | 0.002 |
| No | 158/261 | 24/3 | 13.22 (3.92-44.6) |  |
| Yes | 217/211 | 32/13 | 2.39 (1.22-4.69) |  |
| Physical inactivity |  |  |  | 0.215 |
| No | 276/371 | 37/11 | 4.52 (2.27-9.02) |  |
| Yes | 94/98 | 18/5 | 3.75 (1.34-10.52) |  |
| Migraine with aura |  |  |  | 0.373 |
| No | 209/397 | 38/13 | 5.55 (2.89-10.65) |  |
| Yes | 166/78 | 18/3 | 2.82 (0.81-9.86) |  |
| Use of estrogen† |  |  |  | 0.917 |
| No | 132/185 | 17/1 | 23.83 (3.13-181.25) |  |
| Yes | 41/41 | 10/0 | 11.00 (1.36-89.15) |  |

Data are number of patients with cryptogenic ischemic stroke/number of controls; aCL, anticardiolipin antibodies.

OR, odds ratio; CI, confidence interval.

*Probability value from logistic regression model, adjusted for age, hypertension, diabetes mellitus, current tobacco smoking, physical inactivity, excessive alcohol use, unhealthy diet, stress, depression, and migraine with aura.

†Only female study subjects were considered.

**Table S9. Univariable Analyses of the Association Between Antiphospholipid Antibodies and Cryptogenic Ischemic Stroke Among Patients Diagnosed With High-Risk Patent Foramen Ovale (PFO).**

| **Antiphospholipid Antibody** | **High-Risk PFO** | | ***P**** |
| --- | --- | --- | --- |
|  | **No** | **Yes** |  |
| LA | | | 0.509 |
| Negative | 239 (62.4) | 144 (37.6) |  |
| Positive | 27 (57.4) | 20 (42.6) |  |
| aβ2GPI | | | 0.206 |
| Negative | 232 (63.6) | 133 (36.4) |  |
| Positive | 26 (54.2) | 22 (45.8) |  |
| aCL | | | 0.436 |
| Negative | 224 (61.7) | 139 (38.3) |  |
| Borderline or Positive | 35 (67.3) | 17 (32.9) |  |

Data are n (%). LA, Lupus Anticoagulant; aβ2GPI, anti-β2-glycoprotein I antibodies; aCL, anticardiolipin antibodies; OR, odds ratio; CI, confidence interval.

*Unadjusted P-value

**Table S10. List of SECRETO Study Centers Enrolling Participants and Investigators Involved (PI, Principal Investigator).**

| **Country** | **Center** | **Investigators (alphabetic order)** |
| --- | --- | --- |
| Estonia | Tartu University Hospital, Tartu | Karu Külliki  Kõrv Janika, PI  Kõrv Liisa  Muda Piibe  Vibo Riina |
| Finland | Helsinki University Hospital, Helsinki | Artto Ville  Curtze Sami  Gordin Daniel  Groop Per-Henrik  Haapalahti Petri  Jalanko Mikko  Lehto Mika  Martinez-Majander Nicolas  Pirinen Jani  Putaala Jukka, PI  Rapola Janne  Sairanen Tiina  Sibolt Gerli  Sinisalo Juha  Soinne Lauri  Strbian Daniel  Suihko Satu  Tiainen Marjaana  Tolppanen Heli  Tulkki Lauri  Tuohinen Suvi |
| Finland | Kuopio University Hospital and University of Eastern Finland, Kuopio | Autere Jaana  Hedman Marja  Jaakonmäki Nina  Jäkälä Pekka, PI  Kantanen Anne-Mari  Miettinen Tuuli  Nerg Ossi  Onatsu Juha |
| Finland | Tampere University Hospital, Tampere | Numminen Heikki  Ryödi Essi  Sarkanen Tomi, PI  Virtanen Marko |
| Finland | Turku University Hospital and University of Turku, Turku | Lautamäki Riitta  Roine Risto O.  Saraste Antti  Ylikotila Pauli, PI |
| Finland | Oulu University Hospital, Oulu | Huhtakangas Juha, PI  Junttola Ulla  Keskinarkaus Irma |
| Germany | University Medical Greifswald, Greifswald | Busch Raila  Holbe Christine  Schminke Ulf  von Sarnowski Bettina, PI |
| Greece | Attikon University Hospital, Athens | Frogoudaki Alexandra  Papadimitripoulos Georgios  Tsivgoulis Georgios, PI |
| Italy | Azienda Unità Sanitaria Locale - IRCCS, Reggio Emilia | Grimaldi Teresa  Malferrari Giovanni  Zedde Marialuisa, PI |
| Italy | University of Brescia, Brescia | Filomena Caria  Lombardi Carlo Maria  Pezzini Alessandro, PI  Poli Loris |
| Lithuania | Vilnius University Hospital, Vilnius | Jatuzis Dalius, PI  Kramena Rita  Masiliunas Rytis  Ryliskiene Kristina  Zakarkaite Diana |
| Netherlands | Radboud University Medical Center, Nijmegen | de Leeuw Frank-Erik, PI  Ekker Merel  Elias-Smale Suzette  van Dongen Myrna M.E. |
| Norway | Haukeland University Hospital, Bergen | Fromm Annette  Gerdts Eva  Naess Halvor  Saeed Sahrai  Waje-Andreassen Ulrike, PI |
| Portugal | Hospital de Santa Maria, University of Lisbon, Lisbon | Almeida Ana  Amorim Isabel  Ferro José Manuel  Fonseca Ana Catarina, PI |
| Spain | Torrecardenas University Hospital, Spain | Martinez Sanchez Patricia, PI  Mejias Olmedo Victoria  Pascasio Laura Amaya |
| Sweden | Sahlgrenska Academy at University of Gothenburg and Sahlgrenska University Hospital, Gothenburg | Abrahamson Margareta  Bech-Hanssen Odd  Davidson Maria  Holmegaard Lukas  Jerndal Mikael  Jood Katarina  Nordanstig Annika  Redfors Petra, PI  Tatlisumak Turgut |
| Turkey | Istanbul University, Istanbul | Ekizoglu Turgut Esme  Elitok Ali  Sonsöz Mehmet  Yesilot Nilufer, PI |
| United Kingdom | Royal Stoke University Hospital, Stoke-on-Trent | Ferdinand Phillip, PI  Oxley Cheryl  Pencz Zoltan  Roffe Christine |
| United Kingdom | Peterborough City Hospital, Peterborough | Hacque Mohammad  Licenik Radim, PI  Owusu-Agyei Peter  Subramonian Santhosh |

**Supplement References**

1. Martinez-Majander N, Artto V, Ylikotila P, et al. Association between migraine and cryptogenic ischemic stroke in young adults. *Ann Neurol*. 2021;89:242-253.

2. Craig C, Marshall A, Sjöström M, et al. International physical activity questionnaire: 12-country reliability and validity. *Med Sci Sports Exerc*. 2003;35:1381-1395.

3. Martinez-Majander N, Kutal S, Ylikotila P, et al. Association between heavy alcohol consumption and cryptogenic ischaemic stroke in young adults: a case-control study. J *Neurol Neurosurg Psychiatry.* 2024: 333759

4. Panagiotakos Db, Pitsavos C, Arvaniti F, et al. Adherence to the Mediterranean food pattern predicts the prevalence of hypertension, hypercholesterolemia, diabetes and obesity, among healthy adults; the accuracy of the MedDietScore. *Prev Med*. 2007;44:335‑340.

5. Jaakonmäki, N., Zedde, Ml., Sarkanen, T, et al. Obesity and the Risk of Cryptogenic Ischemic Stroke in Young Adults. *J Stroke Cerebrovasc Dis*. 2022;31:106380

6. WHO Nutrition and Food Safety Team. Waist circumference and waist-hip ratio: report of a WHO expert consultation. World Health Organization. <https://www.who.int/publications/i/item/9789241501491>. 2008. Accessed February 9, 2021.

7. Radico F, Foglietta M, Di Fulvio M, et al, The ‘dreaded PFO’: anatomical and functional features of high risk for stroke, European Heart Journal Supplements, 2021;23:E189–E193.
